# Association of Texas Senate Bill 8 With Requests for Self-managed Medication Abortion

**DOI:** 10.1001/jamanetworkopen.2022.1122

**Published:** 2022-02-25

**Authors:** Abigail R. A. Aiken, Jennifer E. Starling, James G. Scott, Rebecca Gomperts

**Affiliations:** 1 Lyndon B. Johnson School of Public Affairs, University of Texas at Austin; 2 Mathematica Policy Research Inc, Cambridge, Massachusetts; 3 Department of Statistics and Data Sciences, University of Texas at Austin; 4 Women on Web, Amsterdam, the Netherlands

## Abstract

This cross-sectional study examines whether the passage of Texas Senate Bill 8 was associated with an increase in requests for self-managed medication abortion.

## Introduction

On September 1, 2021, Senate Bill (SB) 8 went into effect in Texas. The law bans abortion with almost no exceptions after approximately 6 weeks of pregnancy,^1^ raising the question of whether more people in Texas sought to self-manage their abortions outside the formal health care setting. Since 2018, nonprofit service Aid Access has been providing self-managed medication abortion through online telemedicine in the US.^2^ The service operates outside the formal health care setting and is accessed through an online consultation form. A donation of $110 is requested but reduced amounts are accepted. Mifepristone and misoprostol are mailed to the requestor for home use, and an online help desk team is available for further information and support.

## Methods

This cross-sectional study analyzes a data set provided by Aid Access containing the date and state of origin of requests between October 1, 2020, and December 31, 2021, to assess whether requests from Texas increased after SB 8 went into effect. The University of Texas at Austin institutional review board approved the study. All those who made a request to Aid Access consented to the fully deidentified use of their data for research purposes at the time of making the request. This study followed the Strengthening the Reporting of Observational Studies in Epidemiology (STROBE) reporting guideline. We examined the absolute and percentage change in mean daily requests before and after SB 8, using 2-sample *t* tests with unequal variances to assess significance, which was set at *P* = .05. Data were analyzed on a rolling basis between September 2021 and January 2022. We used R statistical software version 3.5.3 (R Project for Statistical Computing) for data analysis.

## Results

Between October 1, 2020, and December 31, 2021, Aid Access received 45 908 requests for medication abortion from all 50 US states. Between October 1, 2020, and May 9, 2021, there was a mean (SD) of 10.8 (3.7) requests per day to Aid Access from Texas (Figure, panel B). A small increase occurred in mid-May, when SB 8 was returned from the House for final passage (Figure, panel A). Then, in the first week after SB 8 went into effect (September 1-8, 2021), mean (SD) daily requests increased by 1180% over baseline, from 10.8 (3.7) to 137.7 (85.7) requests per day (Figure, panel B) (95% CI, = 47.7-206.2; *P* = .008). During the subsequent 3 weeks (September 9-30, 2021), requests decreased from their peak, but remained 245% higher than the pre–SB 8 baseline at a mean (SD) of 37.1 (9.1) vs 10.8 (3.7) requests per day (Figure, panel B) (95% CI, = 22.4-30.27; *P* < .001). Overall, Aid Access received 1831 requests from Texas for self-managed abortion in September 2021. Over the following 3 months (October 1 to December 31, 2021), there was a mean (SD) of 29.5 (8.2) requests per month, 174% higher than the pre–SB 8 baseline (Figure, panel B) (95% CI, = 17.0-20.5; *P* < .001). During the same periods, mean daily requests from the other 49 US states showed much smaller increases (Figure, panel C), perhaps because of increased awareness of the service and other restrictive legislation.

**Figure.**
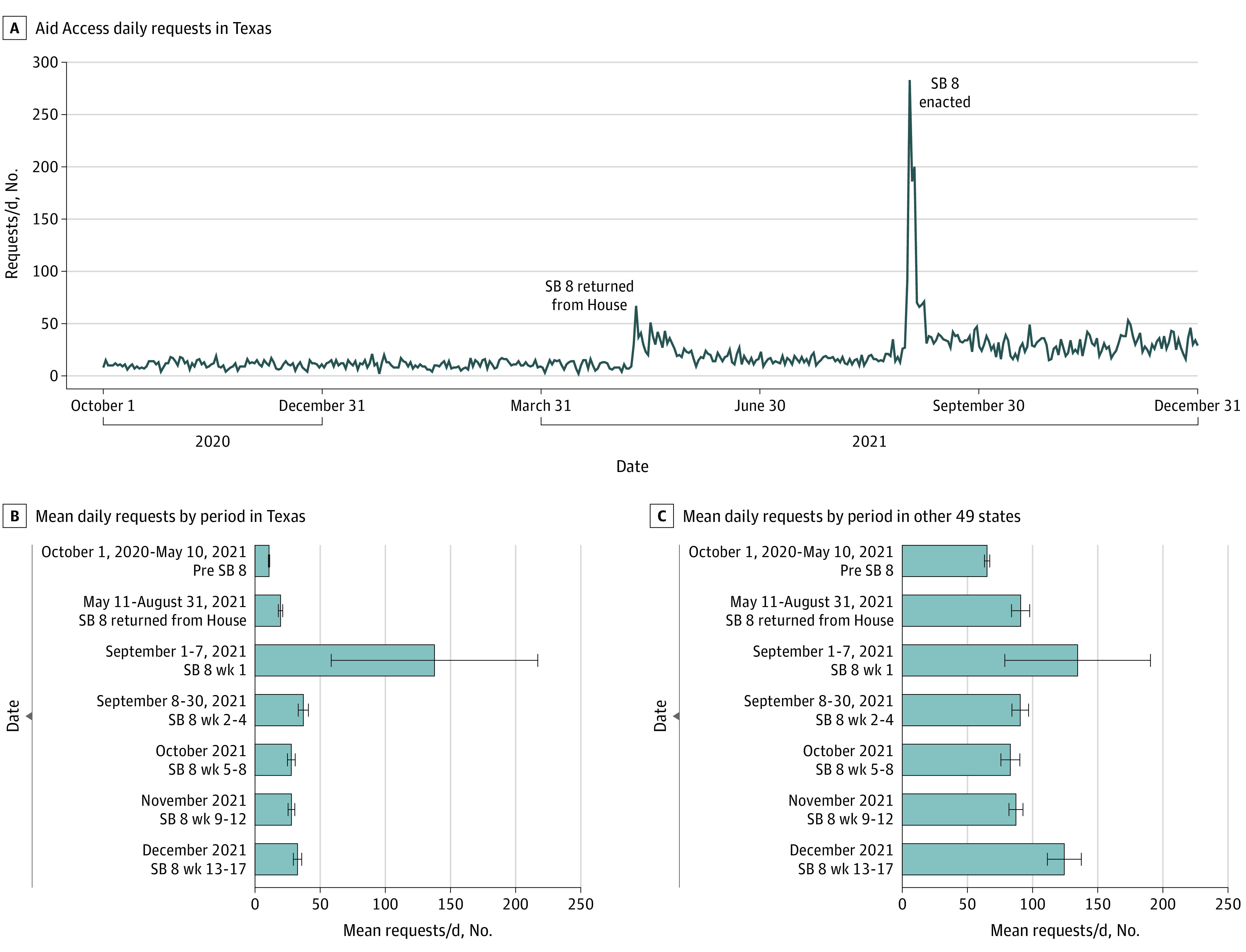
Requests to Aid Access Between October 1, 2020, and December 31, 2021 Panel A shows daily requests to Aid Access from Texas, and panels B and C show the mean request rate before and after Senate Bill (SB) 8 took effect in Texas and in the other 49 US states, respectively.

## Discussion

The findings of this cross-sectional study show that after SB 8 went into effect, demand for self-managed abortion through Aid Access increased substantially in Texas. An initial high increase then leveled off to a more moderate but sustained increase over pre–SB 8 levels. Although we cannot pinpoint the exact reason for this distinctive pattern, uncertainty about eligibility and clinic appointment cancellations may have been associated with the peak increase, whereas grassroots abortion funds and clinics connecting Texans with care out of state likely were associated with the subsequent decrease.^3^ SB 8 may also have primed people to recognize their pregnancies earlier, resulting in more abortions at less than 6 weeks’ gestation at Texas clinics. A limitation of the study is that we cannot determine whether all requests resulted in abortions. We also cannot determine the prevalence of self-management using other methods.

Overall, Aid Access received 1831 requests from Texas for self-managed abortion in September 2021. To put this figure in perspective, 4511 abortions were conducted in Texas clinics in September 2020,^4^ vs 2164 in September 2021,^5^ a year-on-year decrease of 2347 abortions. Given that clinics in neighboring states and even in states that do not border Texas have reported an increased volume of patients from Texas,^6^ it seems likely that in the face of uncertain options, some people made requests to Aid Access as well as seeking in-clinic care. Self-managed abortion may, therefore, provide an important back-up option, as well as a pathway to care for those ultimately unable to access a clinic.
