# Error in Results

**DOI:** 10.1001/jamanetworkopen.2022.7184

**Published:** 2022-03-21

In the Research Letter titled “Association of Texas Senate Bill 8 With Requests for Self-managed Medication Abortion,”^1^ published February 25, 2022, there was an error in the Results. The mean (SD) number of requests should have read 29.5 (8.2) per day. This article has been corrected.^1^
